# Patient Perspectives on Post-Discharge Surgical Site Infections: Towards a Patient-Centered Mobile Health Solution

**DOI:** 10.1371/journal.pone.0114016

**Published:** 2014-12-01

**Authors:** Patrick C. Sanger, Andrea Hartzler, Sarah M. Han, Cheryl A. L. Armstrong, Mark R. Stewart, Ross J. Lordon, William B. Lober, Heather L. Evans

**Affiliations:** 1 Department of Biomedical Informatics & Medical Education, University of Washington, Seattle, Washington, United States of America; 2 Group Health Research Institute, Group Health Cooperative, Seattle, Washington, United States of America; 3 Department of Surgery, University of Washington, Seattle, Washington, United States of America; 4 Department of Biobehavioral Nursing and Health Systems, University of Washington, Seattle, Washington, United States of America; ISMETT-UPMC Italy/University of Catania, Italy

## Abstract

**Background:**

Post-discharge surgical site infections (SSI) are a major source of morbidity, expense and anxiety for patients. However, patient perceptions about barriers experienced while seeking care for post-discharge SSI have not been assessed in depth. We explored patient experience of SSI and openness to a mobile health (mHealth) wound monitoring “app” as a novel solution to address this problem.

**Methods:**

Mixed method design with semi-structured interviews and surveys. Participants were patients who had post-discharge surgical wound complications after undergoing operations with high risk of SSI, including open colorectal or ventral hernia repair surgery. The study was conducted at two affiliated teaching hospitals, including an academic medical center and a level 1 trauma center.

**Results:**

From interviews with 13 patients, we identified 3 major challenges that impact patients' ability to manage post-discharge surgical wound complications, including required knowledge for wound monitoring from discharge teaching, self-efficacy for wound monitoring at home, and accessible communication with their providers about wound concerns. Patients found an mHealth wound monitoring application highly acceptable and articulated its potential to provide more frequent, thorough, and convenient follow-up that could reduce post-discharge anxiety compared to the current practice. Major concerns with mHealth wound monitoring were lack of timely response from providers and inaccessibility due to either lack of an appropriate device or usability challenges.

**Conclusions:**

Our findings reveal gaps and frustrations with post-discharge care after surgery which could negatively impact clinical outcomes and quality of life. To address these issues, we are developing mPOWEr, a patient-centered mHealth wound monitoring application for patients and providers to collaboratively bridge the care transition between hospital and home.

## Introduction

Surgical site infections (SSI) occur in 3–5% of all surgical patients, and up to 33% of patients undergoing abdominal surgery [1], [2]. With shorter hospitalizations, most SSIs now occur post-discharge, placing a burden on patients who are often ill-prepared to manage SSI [3] – [6]. More than half of patients who develop post-discharge SSI are readmitted to the hospital, making SSI the overall costliest healthcare-associated infection [5], [7], [8]. Non-financial costs of post-discharge SSIs to patients are also high, especially in decreased quality of life [9].

The transition between in-hospital and post-discharge surgical care poses special challenges that exacerbate the morbidity of post-discharge SSI. Patients experience a “voltage drop” at discharge—a sudden decrease in supervised wound assessment and patient-provider communication—yet take on primary responsibility for problem recognition and wound care at home [10]. Patients may have minimal or ineffective discharge teaching [11], resulting in lack of knowledge and awareness about SSI, and ultimately, an inability to recognize when an infection develops [12], [13]. Recent studies suggest that inadequate post-discharge communication and untimely, infrequent follow-up contribute to poorer outcomes (e.g. readmission) [14], [15].

Mobile health (mHealth) may present an opportunity to improve the identification and management of post-discharge SSI. Smartphones possess high quality cameras and constant internet connectivity, providing an ideal platform for multimedia clinical data collection and real-time patient-provider communication [16]. Patients are increasingly interested in and equipped to manage their health with technology, with 56% of US adults owning a smartphone and 69% tracking at least one health indicator [17], [18]. To better understand how patients experience SSI following surgery and how technology could improve their experience, we addressed the following hypothesis-generating research questions:

1. What challenges do patients experience when identifying and managing surgical wound complications after discharge?
2. What are patients' perceptions about the acceptability of an mHealth wound monitoring application to address those challenges?

## Methods

We conducted a mixed-methods study comprised of semi-structured interviews and surveys with patients who experienced surgical wound complications after hospital discharge.

### Ethics statement

The study was approved by the University of Washington Institutional Review Board and written consent was obtained from all participants prior to undergoing study procedures.

### Participants and setting

We interviewed adult, English-speaking patients who had post-discharge wound complications after undergoing an intra-abdominal operation at high risk for SSI. Patients were recruited at two University of Washington general surgery clinics. Following the standard discharge protocol, patients were asked to call the clinic if they had concerns prior to their follow-up visit, which generally occurred 1–2 weeks post-discharge.

Using consecutive sampling, patients were identified either directly by clinic nurses at follow-up visits or through patient-initiated contact via IRB-approved recruitment flyers placed in surgery clinics.

### Data collection

We conducted one-on-one, semi-structured interviews lasting 60–90 minutes in a private setting near the clinics. The interview consisted of two parts (session guide: Appendix S1). First, to understand challenges patients face managing post-discharge wound complications, we used the critical incident technique to guide patients in recounting their wound complication experience [19]. Second, to understand patients' perceptions about the acceptability of mHealth for post-discharge wound monitoring and care coordination, we introduced paper mockups of a wound monitoring application that illustrated key features: symptom tracking, wound photography, secure communication, and informational content. Participants then responded to multiple-choice and open-ended survey questions about the acceptability of such mHealth for wound monitoring, level of technology experience (adapted from national surveys [17], [18]), and demographics. Interviews were audio recorded and transcribed. Subject accrual continued until thematic saturation was achieved (i.e. no new data or themes were encountered) [20].

### Data analysis

We used a grounded theory approach to data analysis, not relying on pre-determined codes or coding schemes [20]. The initial four transcripts were each independently coded by 4 members of the research team. The group then discussed and recoded the transcripts in concert, allowing key themes to emerge in an inductive manner. We collectively developed a consensus codebook (Appendix S2) which two team members (PCS, SMH) used to code all interviews using Atlas.ti (Atlas.ti v7, ATLAS.ti GmbH). Other team members spot-coded interviews to inform the codebook and check coding reliability. The team met periodically to resolve coding discrepancies. Cohen's Kappa between the two primary coders during early and late coding was 0.51 and 0.71, respectively, reflecting moderate to substantial inter-coder reliability [21]. Descriptive statistics from surveys were calculated with Microsoft Excel.

## Results

Interviews revealed unique patient insights into the SSI experience, including challenges faced and how an mHealth solution could address those challenges. After describing our participant sample, we detail 3 major themes that emerged about barriers and facilitators that patients experience while managing surgical wounds after discharge. We then summarize participant acceptability of mHealth for post-discharge wound management, including perceived benefits and limitations. Dataset S1 and Dataset S2 contain additional quotes from patients and survey results, respectively.

### Participants

Over 4 months, we identified 17 consecutive adult patients who experienced post-discharge wound infection following abdominal surgery. Of the 17 eligible patients, 13 participated (i.e., P1–P13). The remaining 4 were willing to participate, but either faced time constraints (n = 2) or had psychiatric illness (n = 2).

We report participant demographics in Table 1 and technology experience in Table 2. Participants described the duration of their wound problems lasting up to weeks or months after discharge, and 5 reported one or more emergency department visits or hospital readmissions related to SSI.

**Table 1:** Participant demographics.

Age	
Mean [SD]	45 [15]
Median [range]	51 [21]–[71]
Gender	
Female	9 (69%)
Adults in household	
1	4 (31%)
2	4 (31%)
3+	5 (38%)
Race	
American Indian	1 (8%)
Asian	2 (15%)
White	9 (69%)
Other	1 (8%)
Education	
Less than high school	1 (8%)
High school graduate	1 (8%)
Some college	6 (46%)
College graduate	5 (38%)

**Table 2:** Participant technology experience.

Experience with computers	
Some experience	3 (23%)
Intermediate	4 (31%)
Very experienced	4 (31%)
Expert	2 (15%)
Devices currently owned *	
Desktop computer	8 (62%)
Laptop computer	11 (85%)
Smartphone	8 (62%)
Tablet	6 (46%)
Cellphone	12 (92%)
Internet use *	
At least occasional use (any device)	12 (92%)
Any use on cellphone or tablet	8 (62%)
Primary use on cellphone	3 (23%)
Use of cellphone to… *	
Send or receive email	6 (46%)
Send or receive text messages	9 (69%)
Take a picture	10 (77%)
Download software or “app”	6 (46%)
Use health “apps”	2 (15%)
None of the above	2 (15%)

Asterisk (*) indicates percentages not summing to 100%. Participants could indicate one or more answers to these questions.

### Challenges of coping with wound complications

**Figure 1:**
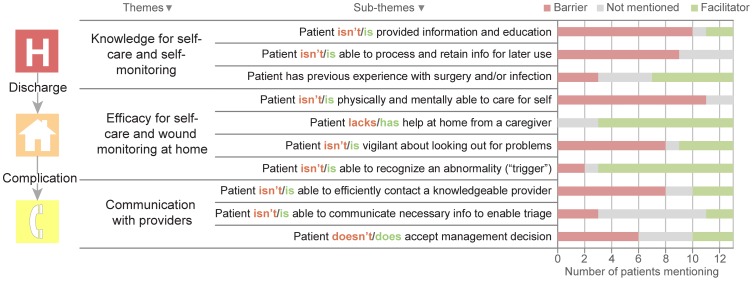
Barriers and facilitators to coping with wound complications. Ten sub-themes identified from patient interviews related to coping with post-discharge wound complications, organized into 3 major themes. The color distribution of each bar represents the number of participants who considered each sub-theme to be a barrier (red) or a facilitator (green). Grey indicates that the participant did not mention the sub-theme.

Three major themes emerged from interviews on patient self-management of post-discharge wound complications: knowledge for self-care and self-monitoring, efficacy for self-care and wound monitoring at home, and communication with providers (Figure 1). Each theme is comprised of 3 or more sub-themes (i.e., “codes”) that emerged from our analysis. Although most of the 10 sub-themes are primarily barriers (e.g., poor physical or cognitive state limiting self-care) or facilitators (e.g., help at home from a caregiver), some sub-themes serve as either a barrier or facilitator, depending on the situation (e.g., previous experience with surgery). We organized these 10 sub-themes into 3 broader themes, detailed next.

#### Knowledge for self-care and self-monitoring

The first theme relates to the quality of discharge teaching participants received (e.g., handouts/resources patients are provided) and the challenge of processing, retaining and using that information. This theme also reflects differences in prior knowledge from previous surgeries, which affect patients' information needs.

Ten participants reported not receiving adequate information or education during the discharge process. They attributed their anxiety and some wound complications to this failure to teach appropriate management and monitoring skills before going home. They also mentioned a lack of reference material for use at home and material that was too generic.

*“Well, I think [the infection] developed because I wasn't changing the dressing. I wasn't made aware of how to do that.” *(P1)

*“*[My experience was] *horrible. You know, I didn't have a clue what to do. I called a friend who had taken care of people in hospice or I wouldn't have known that I had to pack a wound – they didn't say what to do, they just gave me all that stuff and said ‘here'.”* (P2)

In addition to not receiving the desired information, nine participants noted their inability to process and retain the information that was provided. At the time of their discharge teaching, participants reported having pain, feeling mentally slowed and disoriented by pain medications, and overwhelmed by the amount of information they had to take in. Many participants also felt that the discharge teaching did not suit their learning preference (e.g., written instructions versus hands-on experience).

*“It [wound care instructions] might have been on paper, you know, kind of trying to explain it, but I didn't have a clue what to do with it and I had to call a friend who did know.” *(P2)

*“I think probably I forgot a lot of what people told me… because I was very concerned about my shoulder [other injury], and also I was really drugged up.” *(P7)

Nine participants had experienced a previous surgery or infection. For 6 participants, this experience was a facilitator – they reported increased confidence, required less information, and were more active in monitoring their surgical wound. Conversely, 3 patients had uncomplicated prior surgeries, leading to less engagement than in their previous experience.

*“I've been in the hospital a lot. I mean I've been answering doctors' and nurses' questions for years, so I kind of know better what they are looking for and what I should be looking for.” *(P10)

*“No [I wasn't concerned about infection]. Because I've had four other surgeries, five other surgeries and never had an issue with any of them.” *(P6)

#### Efficacy for self-care and wound monitoring at home

The second theme relates to challenges patients face, often with the help of caregivers, in effectively caring for themselves. In particular, this included being vigilant for wound problems, and recognizing wound problems when they surface.

After leaving the hospital, 11 participants reported that they were physically and/or cognitively incapable of caring for themselves. The most commonly reported barriers to self-care included medication use, pain, and feeling overwhelmed or squeamish about wound care. They reported that their poor state lead to decreased vigilance, less information-seeking, diminished self-care efficacy, and increased need for home assistance.

*“I may have normally [sought information about wound care], but I was taking a fair amount of probably – was it Oxycodone? – so no, I didn't really think of that (laughs).” *(P8)

*“I thought it was real early to have been discharged. … I could barely walk and I couldn't hold my pee and I wasn't normal at all… and weak and out of it.” *(P2)

Ten participants reported receiving help from a caregiver at home (e.g., spouse, friend, family member). Participants noted that lack of information about wound care, poor physical/cognitive state, and physical limitations due to surgery (e.g., inability to reach surgical site) contributed to their need for additional support at home.

*“He [my husband] was helping me change the dressing, because I was not feeling real good. I mean I was not sleeping real well. I had a lot of pain from the surgery itself.” *(P8)

*“Not everybody has somebody at home that can be there twice a day… I couldn't have done it myself. There's no way.” *(P13)

Eight participants reported a lack of vigilance about wound infection—i.e. they were not actively looking out for problems. Other participants demonstrated vigilance by closely following symptom trends or sending wound photos to providers to make sure they were healing appropriately (wound photos discussed further under “Communication with providers” below).

*“No… my main concern was the weakness and the pain. I didn't really think about infection. Maybe I should have.” *(P2)

*“There is an awful lot of people out there, I'm one of them, that says oh no, it's nothing to worry about, this will get better. I'm not going to complain.” *(P12)

Although many participants did not actively look for wound problems, 10 recognized when a problem surfaced, often stemming from a sense that ‘something wasn't right'. In other cases participants did not have a typical symptom or know that their symptom was abnormal.

*“Because I was taking pain medications and even so I started feeling pain, so that's what made me concerned. Because it wasn't getting better, it was getting worse.” *(P5)

*“It took a long time to heal, and it oozed a lot… I thought it was normal… I didn't know that other people didn't have it, didn't have a clue. I didn't know till today [follow-up appointment] I had an infection.” *(P2)

#### Communication with providers

The third theme relates to the challenges patients face efficiently contacting providers about wound concerns, providing necessary information for triage, and then working with providers to arrive at an acceptable management decision.

After developing a wound concern, 8 participants had trouble reaching a provider who was familiar with their case. Participants reported problems contacting providers after hours or on weekends, getting *‘*the run around' trying to talk to the right person, not knowing who to contact, frustration with leaving messages, and even putting off care concerns until business hours. Some participants developed a routine to communicate with a specific nurse, received a direct-access number, or made use of photos, text messages, or email – all of which tended to decrease anxiety.

*“Noticed it [infection] on Sunday, waited because I didn't want to have to go to the ER until I could talk to a nurse … I called the number and then I got put on hold and then run through like three different people before I finally got to a nurse.” *(P6)

*“First I called the nurse's hotline or whatever. And I talked to them, and it was hard to get a hold of anyone who even knew what was going on with my case or anything.” *(P7)

Two participants sent wound photos to their providers (one patient-initiated, one provider-initiated) to more fully communicate their situation. Participants appreciated sending photos instead of trying to explain in words alone, and hoped that photos might prevent unnecessary visits. These two participants were asked to return to clinic early on the basis of their photos.

*“I just sent [the photo]… thought it would just be easier… Instead of just kind of explaining it. Sometimes it's easier with pictures.” *(P4)

*“I thought that was very good to be able to send them an actual picture of what was happening… a little more hands on than ‘okay - this is…' - trying to describe it over the phone… The nurse commented about how good that was too to have a picture to look at.” *(P6)

After contacting providers about their concerns, 6 participants were unhappy with the management of their case, with several feeling unnecessarily shunted to the emergency department. Some participants delayed seeking advice over the weekend to avoid being told to go to the emergency department, while others expressed a desire for more interaction with providers at critical times.

*“And again, same situation – if you're worried about it, go to an emergency room. They never say, well, come on up and we'll check you out.” *(P8)

*“I contacted them and they said well, you have an appointment here in a few days. Let's just wait it out and see… I felt a little put off. Like their sense of urgency for me wasn't really there.” *(P13)

### Acceptability, perceived benefits, and potential limitations of an mHealth solution

**Figure 2:**
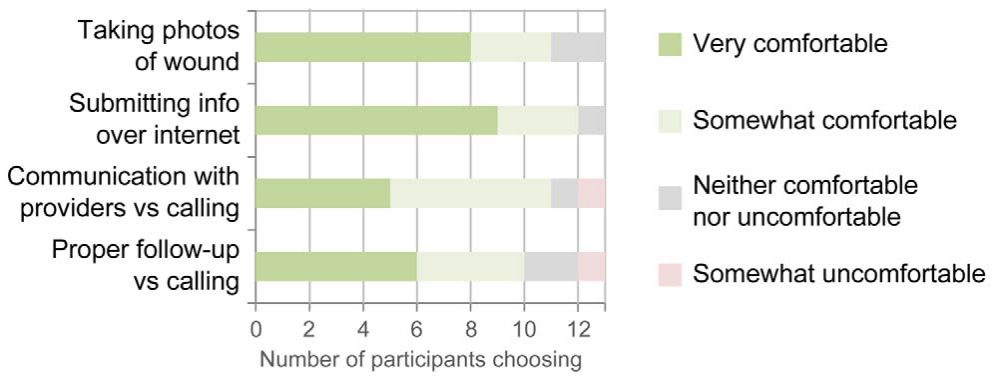
Comfort with mHealth application. Participants' comfort with 4 key elements of a wound-tracking mHealth application. Based on 4 survey questions, participants were either “Very comfortable” (dark green), “Somewhat comfortable” (light green), “Neither comfortable nor uncomfortable (grey), or “Somewhat uncomfortable” (light red). No participants selected “Very uncomfortable”.

After reviewing paper mockups of an mHealth wound monitoring application, participants expressed broad comfort with its key features and trusted that it could facilitate proper follow-up (Figure 2). Table 3 shows the top 4 perceived benefits and limitations participants attributed to the application.

**Table 3:** Perceived benefits and limitations of mHealth approach.

	N	Exemplary quote
**Perceived Benefits**		
Easier/more frequent follow-up	4	*“Easier/more frequent follow-up especially just after discharge from hospital.”* **P3**
Better triage (e.g., fewer ED visits)	4	*“It would save money for both patient and healthcare facility, and it would save the patient from unnecessary trips to the ER or clinic.”* **P12**
Less anxiety	4	*“I think this system would promote feelings of relief… that the doctor/staff is aware of your situation and that you are being taken care of without having to go in to the office unless you have to. Peace of mind.”* **P10**
Photos: clearer, easier, fuller communication	3	*“Having logged data and pictures seems better than trying to explain what's been going on for the past week or so in words to your doctor.”* **P5**
**Perceived Limitations**		
Untimely response	4	*“I am a bit concerned with the initial turnaround on responses* [from providers]. *This would require individual providers to embrace the technology as much as patients.”* **P3**
Inaccessibility	4	*“Older patients might not be as willing to use.”* **P6**
Poor picture/response quality	3	*“Getting people to answer the questions correctly.”* **P12**
Security/misuse	1	*“Possible misuse, not of content, but of address info for crooks to locate someone sick and at home.”* **P9**

Based on two survey questions about benefits and limitations, respectively. N denotes number of participants mentioning the particular benefit/limitation.

Most participants indicated that mHealth would be an acceptable solution to enable patients to engage in wound monitoring. More specifically, participants perceived that mHealth can address post-discharge challenges by allowing more frequent, thorough, and convenient follow-up, thus leading to less patient anxiety and fewer unnecessary emergency department visits than current practice. Participants were concerned about lack of timely response from providers and inability to use the application, either due to lack of an appropriate device or difficulty using the application itself.

## Discussion

Post-discharge SSIs are not only a major healthcare quality and cost problem—they are also a significant burden on patients that highlight larger failings in post-discharge care coordination [9], [10], [12]. Our findings reveal frustration with gaps in care that leave patients feeling disconnected from their providers at a critical time in their recovery. Concordant with a previous quantitative study of post-discharge SSI [9], our participants described major impacts of infection on their quality of life, both physically (e.g. due to pain or frequent fluid leakage, or numerous trips to hospital) and emotionally (e.g. due to anxiety related to initial identification of SSI including unsatisfactory attempts to contact providers). In our previous work, providers identified many of the same systemic problems, including challenges communicating prior to scheduled follow-up visits [14].

Historically, patients have not been engaged to prospectively monitor and communicate with providers about their surgical wounds following discharge; typically, surveillance has been passive, retrospective and under the purview of infection control [22]. However, active surveillance programs have been shown to decrease SSI rates [23], [24] and engaged patients have demonstrated improved clinical outcomes and emotional health, and decreased healthcare utilization [25] – [27]. Collecting and analyzing patient-reported outcomes is increasingly recognized as key to engaging patients and providing high-quality, patient-centered care [28].

We believe that an mHealth solution can provide a means to connect patients and providers to enhance patient satisfaction and improve outcomes. As with providers we previously surveyed, patients show openness to an mHealth application for wound self-monitoring, and feel comfortable receiving follow-up through such a system. Patients identified a number of potential strengths and concerns around this mHealth approach, yielding important feedback to inform not only development, but also integration of mHealth applications into care delivery.

Based on our analysis of barriers that patients face when managing post-discharge complications, we suggest that an mHealth wound monitoring application should support enhanced knowledge, self-efficacy, and communication. Such an application could address key barriers to receiving high quality, patient-centered, post-discharge care (Table 4).

**Table 4:** Barriers to post-discharge care addressable by mHealth application.

Barriers	mHealth solutions
Inadequate discharge information	Provide personalized wound care instructions in various multimedia formats accessible before and after discharge by patients and caregivers
Lack of vigilance	Prompt patients to document wounds routinely and support remote monitoring by providers through symptom logs and serial wound photography
Poor communication and sub-optimal management	Contact designated providers familiar with the patient's case by telephone or secure message allowing earlier reassurance or escalation of care

To address these barriers, we are developing an mHealth solution, the Mobile Post-Operative Wound Evaluator (mPOWEr). Informed by user-centered design, this application will facilitate self-monitoring and transmission of clinically actionable serial post-operative wound information, including wound photographs, to surgical providers [29]. Using a dashboard interface, providers will be able to securely monitor data from an individual patient over time, or quickly review data from a panel of patients for prioritization.

Our findings show promise for an mHealth approach, but our exploratory study design has limitations. First, we only interviewed patients who experienced wound complications. Patients without wound problems may have different perceptions about the adequacy of the discharge process or the acceptability of mHealth. To address this limitation, we are currently conducting a prospective survey of recently discharged surgical patients, the majority of whom have not experienced SSI but nonetheless reported high willingness to use a tool like mPOWEr in the future; this study also incorporates a panel of instruments, administered serially, to assess the effect of post-discharge SSI on quality of life. Second, we interviewed a small number of patients from two very different settings within the same local community. As is customary in qualitative research, the sample size was based on reaching saturation (i.e., not hearing new qualitative themes). Despite the small sample, participants were diverse in age, education, and technology experience, and sample characteristics were similar to national samples [17], [18].

Our research has a number of strengths, including diverse perspectives from a multidisciplinary team comprised of a general surgeon, a patient who experienced a post-operative infection, a health informaticist, a user-centered design expert, and a medical student. The qualitative methodological approach enables us to uniquely characterize the post-discharge experience of surgical patients and reveals the need for greater focus on post-discharge care coordination. Finally, our findings form a sound basis for a patient-centered approach to software development, uncommon in the health domain, yet key to developing applications that patients and providers will actually use.

## Conclusions

SSI is a common post-discharge complication and frequently results in readmission and diminished quality of life. In this study, patients who experienced SSI told us that they are not served by the current standard post-hospitalization care practice, reporting deficiencies in discharge education, wound self-monitoring at home, and communication with providers. Patients found the concept of our patient-centered mHealth wound monitoring application (mPOWEr) highly acceptable. Our application will leverage the increasing prevalence of versatile, connected mobile devices for efficient wound monitoring mediated by empowered patients. Future work will focus on the user-centered development of this application and examine its impact on patient satisfaction, quality of life, clinical outcomes, and healthcare utilization.

## Supporting Information

Appendix S1 **Session guide used for patient interviews.**
(PDF)

Appendix S2 **Codebook used to code patient interviews.**
(PDF)

Dataset S1 **Participant quotes illustrating major themes regarding barriers and facilitators to post-discharge surgical wound management.**
(XLSX)

Dataset S2 **Survey data from patients.** From three surveys: application use, technology use, demographics.
(XLSX)
