# Supplementary material for: Patient Perspectives on Post-Discharge Surgical Site Infections: Towards a Patient-Centered Mobile Health Solution
Source: PLoS One. 2014 Dec 1;9(12):e114016. doi: 10.1371/journal.pone.0114016 (PMC4250175; doi:10.1371/journal.pone.0114016)
Supplement: Appendix S1 — Session guide used for patient interviews. (PDF) [file pone.0114016.s001.pdf]

10 minutes

Informed consent

35 minutes

Critical incident review

We will ask questions to better understand how the participant experienced their surgical site infection.

- Tell me about what happened the last time you experienced an SSI….
  - When did you develop your last SSI?
  - How did you initially notice your SSI?
  - What were the most concerning things you noticed?
  - Did your doctors tell you to look for any "warning signs" of infection?
  - Did you know who to call if you suspected an infection?
  - What did you do after you noticed it?
  - How much time elapsed between when you first thought something might be wrong and when you sought medical care?
  - Did you have any family members or other caregivers help you to deal with your SSI?
  - Please describe any interaction with your doctors or other providers.
- Thinking about the time around hospital discharge…
  - Tell me about your follow-up plan, if any, after surgery.
  - If you had a routine follow-up scheduled, how long after discharge was it?
  - Did you feel ready to be discharged?
  - Were you anxious about developing an SSI?
- What was hard about having an SSI?
- What could be done to better support someone who has (or worries they have) an SSI?

15 minutes

Demographic Questions

Technology Use Questions

mPOWEr Use survey
