# Supplementary material for: Patient Perspectives on Post-Discharge Surgical Site Infections: Towards a Patient-Centered Mobile Health Solution
Source: PLoS One. 2014 Dec 1;9(12):e114016. doi: 10.1371/journal.pone.0114016 (PMC4250175; doi:10.1371/journal.pone.0114016)
Supplement: Appendix S2 — Codebook used to code patient interviews. (PDF) [file pone.0114016.s002.pdf]

Visual codebook used by team to code transcripts

Facilitators to SSI detection/patient self-assessment — Illustrative quotes

PrevExperience

"I was told how to take care of the wound. I had done it from a previous surgery, a lot of the wound care was the same, so it was mainly a review."

———

"because I've had this surgery before and I had an infection before, which was way huger than the one that I have now. It was probably like – I had two of them, and it was like maybe three inches each. So I kind of knew kind of what to expect and this one seemed pretty small. So I wasn't shocked or anything about it"

InfoProvided

"they said that if there's any smelly – like smell, if it smelled or fluid came out, to let them know."

———

"And then maybe a couple days later it seemed like a little bit thicker and the site looked a little bit red. So the doctor told me if that stuff happens to call him, so I called him."

InfoProcessed

"yeah, I think at that point they actually opened it up and cleaned it out, at which time somebody went over the process with me again and I realized I think I'd been doing it wrong."

———

"Yeah, I mean because the surgeon, she kind of was doing it while I was watching, so I kind of knew what to do. And it was all right for me. I don't know how other people get through it."

PhysEmotState

"The first time I had help, she did a lot of it, but this time it was so small I can do it myself. And physically I was all right to do it."

———

"I don't know if it's because I have my sleep apnea under control or what the difference was, but the amount of pain and the length of time I was in great pain was much less after the second surgery. So I was in much better shape leaving the hospital."

HomeHelp

"The first time I had help, she [my wife] did a lot of it [self-care], but this time it was so small I can do it myself. And physically I was all right to do it.".

Vigilance

"Right when I noticed it, I contacted my doctor, so actually sent him – took some pictures and I emailed it to him and he just said monitor it. If it – the liquid became thicker or became more red, to let him know. And then it did, so that's when I came in."

———

"Obviously I had a giant gaping wound in my abdomen, I realize infection was a possibility. Also that first time I left the hospital I'd come in with an incarcerated bowel, so I knew infection was a definite possibility."

Trigger

"It actually probably happened about two weeks later when we noticed a little bit of moisture, it actually was leaking on my shirt, that's how I noticed it. And before then, didn't have any problems."

———

"I had no discomfort or anything when I changed the wound, but today, there was redness around the wound itself which was an indication and I do feel some uncomfortableness now."

CommAbleCtc

"Right when I noticed it, I contacted my doctor, so actually sent him – took some pictures and I emailed it to him and he just said monitor it."

———

"No, I mean, [contacting the doctor] was good. He was pretty quick. I figured I'd take pictures so he can visually see it, and he saw it and he kind of made an assessment and told me what to look out for, so he was good."

CommAllInfo

"No, I just sent it, thought it would just be easier that way. Instead of just kind of explaining it. Sometimes it's easier with pictures and stuff."

CommMgmt

"No, I mean, it was good. He was pretty quick. I figured I'd take pictures so he can visually see it, and he saw it and he kind of made an assessment and told me what to look out for, so he was good."

Codes

	PrevExperience	InfoProvided	InfoProcessed	PhysEmotState	HomeHelp	Vigilance	Trigger	CommAbleCtc	CommAllInfo	CommMgmt
**Facilitators** to SSI detection/patient self-assessment	Previous experience as a positive factor	Transition information/help provided (verbally and/or physically)	Information processed/ remembered; evidence of understanding	Good phys./emot. state; can deal with "gross factor"; not compromised by pain; confidence	Has help at home from other caregivers	Self-awareness, self-monitoring, anticipation of issues, "on radar"	Recognizes trigger event; including mentioning of abnormal symptoms	Ability to efficiently contact correct provider **(knowledgeable about pt's case)**	Communication allows conveyance of appropriate information, e.g. wound photos	Communication results in timely, agreeable (to pt) mgmt decisions (incl *not* coming in)
	**Has knowledge for self-care and self-monitoring**			**Has efficacy for self-care and wound monitoring at home**				**Good communication with providers**		
	Pre-existing	*Pre-discharge*		*Pre-concern*				*Post-concern*		
	**Lacks knowledge for self-care and self-monitoring**			**Lacks efficacy for self-care and wound monitoring at home**				**Insufficient communication with providers**		
**Barriers** to SSI detection/patient self-assessment	No previous experience or previous experience as a negative	Transition information/help not provided in a form useful to pt	Information not processed/ remembered ("information overload")	Compromised phys/ emot state; can't deal w gross factor; pain is an issue; lacks confidence	Lacks help at home from other caregivers	Lacks self-awareness, does not self-monitor, does not anticipate issues	Does not have or recognize trigger event	Inability to efficiently contact correct provider	Communication does not allow conveyance of appropriate information	Communication does not result in timely, agreeable management decision; **not getting needed care**
	PrevExperience	InfoProvided	InfoProcessed	PhysEmotState	HomeHelp	Vigilance	Trigger	CommAbleCtc	CommAllInfo	CommMgmt

Barriers to SSI detection/patient self-assessment — Illustrative quotes

PrevExperience

"you know what to look out for, but for someone who hasn't had it before, I don't know if it was enough information or not."

———

"No [I wasn't concerned about developing an infection]. Because I've had four other surgeries, five other surgeries and never had an issue with any of them."

InfoProvided

"You know, I didn't have a clue what to do. That's the basic – I called a friend who had taken care of people in hospice or I wouldn't have known that I had to pack a wound – they didn't say what to do, they just gave me all that stuff and said here. I didn't know you had to wick it in there and – nothing. So he packed it for me for around – I mean, I was kind of out of it for quite a while. I don't know if that's normal, but I figured it was (laughs)."

———

"When they are in the hospital, have a clinic person or whoever is the wound person come through and make sure the patient understands and can overcome any difficulties they have in doing what they have to do at home. That's the priority"

InfoProcessed

"… but again when somebody is going through discharge and you have that much information a lot of it probably isn't going to get through. The standard procedure is a person retains 10%. So if you're going to do a presentation for somebody coming out of the hospital, you should only have the highlights."

———

They – [wound care instructions] might have been on paper, you know, kind of trying to explain it, but I hadn't – I didn't have a clue what to do with it and I looked, and like I said, I had to call a friend who did know. I'm pretty sure there wasn't anything even written that said, you know, pack [the wound] and – they might have thought I knew."

PhysEmotState

"And so I didn't have the strength to overcome my own emotions about doing it. So, it's been about 2 days without any attention. I think that's how the infection started."

———

"I may have normally [sought out resources for wound care], but I was taking a fair amount of probably – was it Oxycodone? – so no, I didn't really think of that (laughs)."

———

" I was out of it. I mean, I was just trying to survive."

HomeHelp

"Well, I could barely walk and I couldn't hold my pee and I mean, you know, I wasn't normal at all. You know, and oozing out and just soaked in the front. And weak and out of it. I didn't – I would have thought that people that were like I was, they'd send them to like a nursing home or something, not that I wanted to go, but you know, see if you had someone to take care of you. Not just send you home by yourself like that."

Vigilance

"No, infections are serious. I didn't even think about that. That could kill you, and you don't even know it, you just get sicker and sicker and – bite it."

———

"No, I was just worried about following instructions. I didn't think of anything else, just change the bandage."

Trigger

"Well, there is always a little bit of pain or discomfort but nothing, just like now with this, I don't feel anything different."

———

"No, I thought [liquid oozing from wound] was normal. Like I said, I thought that they sent you home when it get that good (laughs). I didn't know that other people didn't have it, didn't have a clue. I didn't know till today I had an infection."

CommAbleCtc

"No [I didn't know who to call if I had a problem]. I ended up at the ER, like I said. Nope."

———

"Like this past weekend I called on the phone, I got somebody but I had to run around to get them."

———

"Well, I had a list… if I had medical problems there was a number, if I had appointment problems there was a number… trouble is on Saturday and [Sunday] those numbers aren't manned, so they don't do any good."

CommAllInfo

"probably what would have been helpful would have been a better line of communication for actually seeing the wound and being able to communicate more – being able to better communicate what was going on with the wound so that maybe after things kind of fell into a routine, we wouldn't have needed quite as many follow-ups"

CommMgmt

"Well, some of the secondary infections, the ones that became swollen and eventually burst, especially when the first one kind of appeared and was starting to get painful, I would have definitely have liked to have had a little bit more interaction."

———

The bruise got to be as big as a baseball and I still have a lump there. It's filled up with fluid now. And again, same situation – if you're worried about it, go to an emergency room. They never say, well, come on up and we'll check you out. They never say that!"
